# LY6D-induced macropinocytosis as a survival mechanism of senescent cells

**DOI:** 10.1074/jbc.RA120.013500

**Published:** 2020-11-24

**Authors:** Taiki Nagano, Tetsushi Iwasaki, Kengo Onishi, Yuto Awai, Anju Terachi, Shione Kuwaba, Shota Asano, Ryoko Katasho, Kiyoko Nagai, Akio Nakashima, Ushio Kikkawa, Shinji Kamada

**Affiliations:** 1 Biosignal Research Center, Kobe University, Kobe, Japan; 2 Department of Biology, Graduate School of Science, Kobe University, Kobe, Japan; 3 Department of Biology, Faculty of Science, Kobe University, Kobe, Japan; 4 Department of Bioresource Science, Graduate School of Agricultural Science, Kobe University, Kobe, Japan

**Keywords:** cellular senescence/endocytosis, lipid raft, LY6D, macropinocytosis, Ras protein, vacuole, BSA, bovine serum albumin, EIPA, ethylisopropylamiloride, FTS, farnesyl thiosalicylic acid, GPI, glycosylphosphatidylinositol, LY6D, lymphocyte antigen 6 complex, locus D, 3-MA, 3-methyladenine, MβCD, methyl-β-cyclodextrin, SA-β-Gal, senescence-associated β-galactosidase, SFK, Src family kinases, uPAR, urokinase-type plasminogen activator receptor

## Abstract

Although senescent cells display various morphological changes including vacuole formation, it is still unclear how these processes are regulated. We have recently identified the gene, lymphocyte antigen 6 complex, locus D (LY6D), to be upregulated specifically in senescent cells. LY6D is a glycosylphosphatidylinositol-anchored cell-surface protein whose function remains unknown. Here, we analyzed the functional relationship between LY6D and the senescence processes. We found that overexpression of LY6D induced vacuole formation and knockdown of LY6D suppressed the senescence-associated vacuole formation. The LY6D-induced vacuoles were derived from macropinocytosis, a distinct form of endocytosis. Furthermore, Src family kinases and Ras were found to be recruited to membrane lipid rafts in an LY6D-dependent manner, and inhibition of their activity impaired the LY6D-induced macropinocytosis. Finally, reduction of senescent-cell survival induced by glutamine deprivation was recovered by albumin supplementation to the culture media in an LY6D-dependent manner. Because macropinocytosis acts as an amino acid supply route, these results suggest that LY6D-mediated macropinocytosis contributes to senescent-cell survival through the incorporation of extracellular nutrients.

Cellular senescence is defined as an irreversible cell proliferation arrest induced by various stresses, such as telomere erosion, activated oncogenes, oxidative stress, and DNA damage (1, 2). It has been first described in primary human fibroblasts and now considered to play a critical role in tumor suppression and aging-related diseases including cancer. Senescent cells are known to display a variety of morphological changes including a formation of cytoplasmic vacuoles. However, despite the early discovery of senescence-associated vacuole formation in 1970s (3, 4), it has remained unclear how this process is regulated during senescence and what the physiological significance is.

Lymphocyte antigen 6 complex, locus D (LY6D) is a membrane-bound protein attached to the cell surface via a C-terminal glycosylphosphatidylinositol (GPI) anchor (5). Although LY6D is often used as a surface marker for leukocyte subset identification because of its lineage-specific expression, the physiological function of LY6D is poorly understood. We have recently identified LY6D to be upregulated specifically in senescent cells by comparing the transcriptome between senescent and apoptotic cells and shown that the upregulation of LY6D is dependent on p53, a crucial transcription factor for the initiation and maintenance of senescence (6, 7, 8). However, it is still unknown whether LY6D functionally contributes to the senescence process because our previous study has shown that ectopic expression of LY6D in osteosarcoma U2OS cells had little effect on senescence induction as determined by colony-forming assay and by senescence-associated β-galactosidase (SA-β-Gal) staining, an established marker of senescence.

GPI-anchored proteins are known to show dynamic behaviors in terms of their diffusion, organization, and interactions with other membrane proteins, and at the same time, a certain portion of GPI-anchored proteins is enriched in specialized domains of the plasma membrane to efficiently relay the extracellular signals to intracellular effector molecules (9, 10). These specialized domains, called lipid rafts or microdomains, serve as platforms for dynamic signaling cross talk between GPI-anchored proteins, specific lipids (sphingolipids, etc.), other membrane-associated proteins, and cortical actin filaments. The functional importance of organization of these signaling proteins in the lipid rafts has been demonstrated in several systems, for example, in the activation of Src family kinases (SFK) (11). GPI-anchored proteins, such as CD59, CD73, DAF, and Thy-1, lead to the association and activation of SFK (12, 13, 14). However, in contrast to these GPI-anchored proteins, the functional relevance of LY6D in the activation of intracellular signaling pathways has not been elucidated as yet.

Macropinocytosis is a clathrin- and dynamin-independent endocytic process by which substantial amounts of extracellular fluid and macromolecules are internalized into cells through large irregular vacuoles called macropinosomes (15). Unlike other major endocytic pathways, macropinocytosis is not regulated by cargo-receptor interactions but rather by growth factor–activated tyrosine kinases, such as SFK and downstream Ras-related GTPases. Activation of these signaling molecules drives actin polymerization beneath the cell surface, which in turn promotes actin-mediated membrane ruffling and ultimately induces macropinosome formation. Macropinocytosis has been extensively investigated in macrophages, dendritic cells, and neurons because of its role in sensing the environment through the nonspecific uptake of extracellular fluid. Intriguingly, however, it has been recently reported that the macropinocytic uptake of polypeptides from extracellular fluid allows Ras-transformed cancer cells to survive in conditions where the availability of essential and nonessential amino acids is limited (15, 16). Despite the similarity in high metabolic activity between cancer and senescent cells (2), it has not been investigated whether or not macropinocytosis contributes to survival of senescent cells to date.

In the present study, we explored the relationship between LY6D and the senescence program. We have found that LY6D is needed for the senescence-associated vacuole formation, which occurs through the induction of Ras-mediated macropinocytosis. SFK and Ras were accumulated in the membrane lipid rafts upon senescence in an LY6D-dependent manner. Furthermore, LY6D-induced macropinocytosis can promote survival of senescent cells by the internalization of extracellular fluid under poor nutritional conditions.

## Results

### LY6D induces senescence-associated cytoplasmic vacuole formation

**Figure 1:**
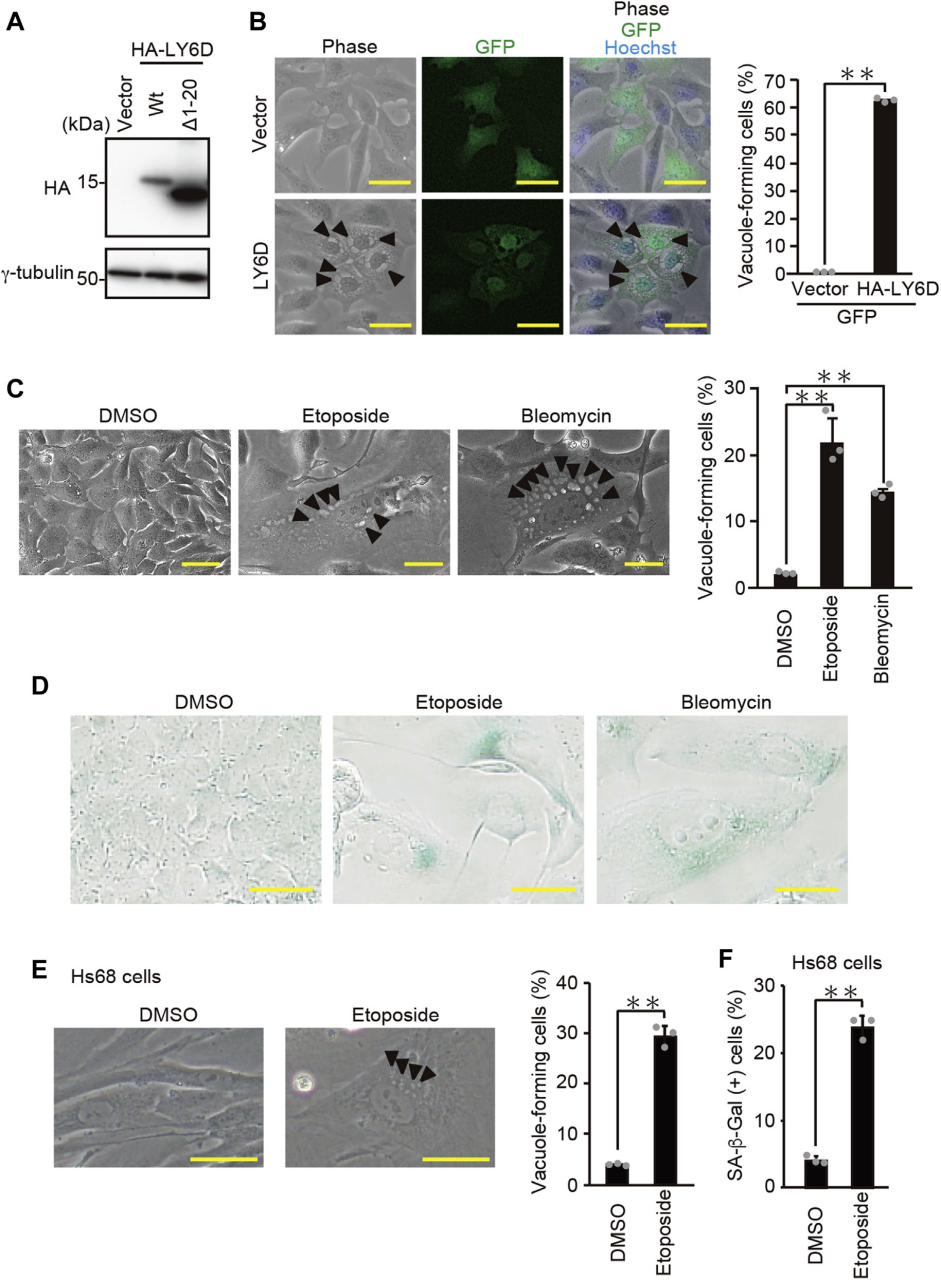
**LY6D induces cytoplasmic vacuole formation, which is the typical morphological change associated with senescence**. *A*, U2OS cells transfected with pcDNA3-HA containing WT and Δ1-20 LY6D were subjected to immunoblot analysis. *B*, U2OS cells cotransfected with pcDNA3-HA-LY6D and pEGFP as a transfection marker for 24 h were observed under microscope. Representative microscopic images (*left*) and the percentage of vacuole-forming cells in GFP-expressing cells (*right*) are shown. *Arrowheads* indicate examples of cytoplasmic vacuoles. *Bars*, 50 μm. *C* and *D*, U2OS cells treated with 2-μM etoposide and bleomycin for 7 days were subjected to observation under microscope (*C*) and SA-β-Gal staining (*D*). *C*, representative microscopic images (*left*) and the percentage of vacuole-forming cells (*right*) are shown. *Arrowheads* indicate examples of cytoplasmic vacuoles. *Bars*, 50 μm. *D*, representative microscopic images are shown. *Bars*, 50 μm. *E* and *F*, Hs68 cells treated with 0.5-μM etoposide for 7 days were subjected to observation under microscope (*E*) and SA-β-Gal staining (*F*). *E*, representative microscopic images (*left*) and the percentage of vacuole-forming cells (*right*) are shown. *Arrowheads* indicate examples of cytoplasmic vacuoles. *Bars*, 50 μm. *F*, the percentage of SA-β-Gal–positive cells is shown. Data are mean ± SD (*n* = 3 independent cultures). Statistical significance is shown using the Student’s *t*-test analysis; ∗∗*p* < 0.01. LY6D, lymphocyte antigen 6 complex, locus D; SA-β-Gal, senescence-associated β-galactosidase.

**Figure 2:**
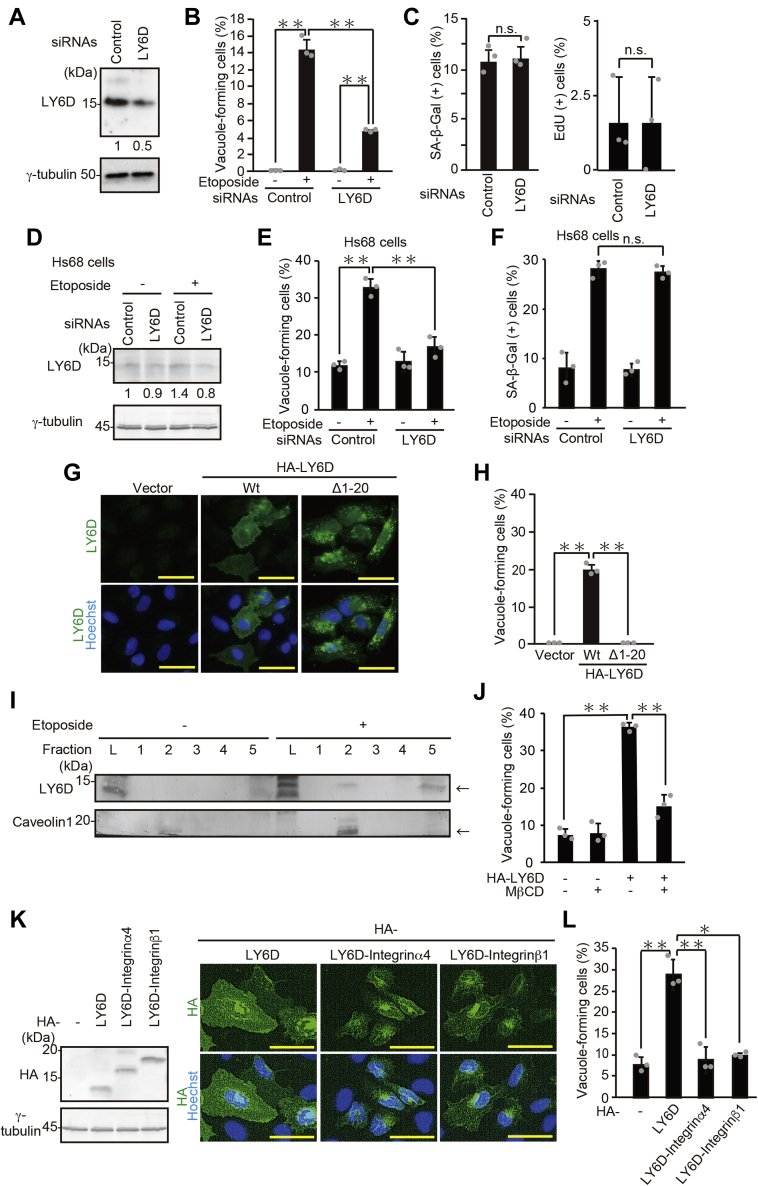
**LY6D induces vacuole formation during senescence, which requires its localization to cell-membrane rafts**. *A*, U2OS cells transfected with siRNA for *LY6D* were subjected to immunoblot analysis. The LY6D protein levels relative to the γ-tubulin levels were quantified using NIH ImageJ software and are indicated at the *bottom* of each lane. *B* and *C*, U2OS cells transfected with siRNA for *LY6D* and treated with 2-μM etoposide for 7 days were subjected to quantification of vacuole-forming cells (*B*) and senescence assays (*C*). The percentage of SA-β-gal–positive cells (C, *left panel*) and EdU-positive cells (*C, right panel*) is shown. *D*-*F*, Hs68 cells transfected with siRNA for *LY6D* and treated with 0.5-μM etoposide for 7 days were subjected to immunoblot analysis (*D*), quantification of vacuole-forming cells (*E*), and SA-β-gal staining (*F*). *D*, the LY6D protein levels relative to the γ-tubulin levels were quantified using NIH ImageJ software and are indicated at the *bottom* of each lane. *G* and *H*, U2OS cells transfected with pcDNA3-HA containing WT and Δ1-20 *LY6D* were subjected to immunostaining with the anti-LY6D antibody (*G*) and quantification of vacuole-forming cells (*H*). *Bars*, 50 μm. *I*, lysates of etoposide-treated U2OS cells were fractionated by sucrose density-gradient centrifugation. The obtained fractions 1 to 5 from the top (light fraction) to the bottom (heavy fraction) of the ultracentrifuge tube were subjected to immunoblot analysis. Caveolin 1 was used as a raft marker, indicating that fraction 2 contained the raft fraction. Fraction L represents whole-cell lysate. The results of different batch experiments are shown in Fig. S6A. *J*, U2OS cells were overexpressed with HA-LY6D-Wt, treated with 2.5-mM MβCD for 17 h, and subjected to quantification of vacuole-forming cells. *K* and *L*, U2OS cells transfected with pcDNA3-HA containing Wt-*LY6D*, *LY6D-integrinα4*, and *LY6D-integrinβ1* were subjected to immunoblot analysis (*K, left*), immunostaining with the anti-HA antibody (*K, right*), and quantification of vacuole-forming cells (*L*). Data are mean ± SD (*n* = 3 independent cultures). Statistical significance is shown using the Student’s *t*-test analysis; ∗*p* < 0.05; ∗∗*p* < 0.01; n.s., not significant (*p* > 0.05). LY6D, lymphocyte antigen 6 complex, locus D; SA-β-Gal, senescence-associated β-galactosidase.

To elucidate the possible function of LY6D in the senescence program, an expression vector containing N-terminal HA-tagged LY6D was constructed and introduced into human osteosarcoma U2OS cells (Fig. 1, *A*; Δ1-20 LY6D mutant lacking N-terminal 20 amino acids was also used, as discussed later). Unexpectedly, we observed the cells showing extensive cytoplasmic vacuole formation after overexpression of LY6D (Fig. 1*B*). The vacuole formation is known as a typical feature of senescence (3, 4). Consistently, treatment of U2OS cells with etoposide and bleomycin, anticancer drugs that cause DNA double-strand breaks, effectively induced the vacuole formation in parallel with the SA-β-Gal activation, an established marker of senescence as measured by X-gal staining where senescent cells were identified as blue-stained cells (Fig. 1, *C* and *D*). Furthermore, the vacuole formation associated with etoposide-induced senescence was also observed in normal human diploid fibroblasts Hs68 cells (Fig. 1, *E* and *F*) in which the *LY6D* gene was previously reported to be upregulated during senescence (6). These results raised the possibility that LY6D is involved in the senescence-associated vacuole formation of both tumor and normal cells. To confirm this, we silenced LY6D by using siRNA in U2OS cells (Fig. 2*A*). Knockdown of LY6D markedly suppressed the vacuole formation during etoposide-induced senescence (Fig. 2*B* and Fig. S1A), whereas it had no effect on both SA-β-Gal activity and cell proliferation capacity (Fig. 2*C* and Fig. S1, *B* and *C*). Similar results were obtained in Hs68 cells (Fig. 2, *D–F* and Fig. S1, *D* and *E*). As confirmed by immunoblotting analysis, the siRNA against *LY6D* inhibited the etoposide-induced upregulation of LY6D in Hs68 cells (compare lanes 3 with 4 in Fig. 2*D*). These results suggest that LY6D is required for the development of senescence-associated vacuoles but not for the induction of senescence itself.

### Localization of LY6D in the membrane lipid raft is required for vacuole formation

Next, we generated an LY6D mutant (Δ1-20 LY6D) harboring a deletion of N-terminal 20 amino acids corresponding to the signal sequence (Fig. 1*A*). As shown in Fig. 2*G*, WT-LY6D was preferentially localized at the cell surface, consistent with the reported localization of LY6D (5), whereas the lack of the signal sequence disrupted the cell-surface localization of LY6D. Furthermore, unlike WT-LY6D, overexpression of Δ1-20 LY6D failed to cause the vacuole formation (Fig. 2*H* and Fig. S1*F*), which leads us to speculate that the membrane-localized LY6D induces the vacuole formation. Because GPI-anchored proteins are known to be distributed to cell membrane lipid rafts/microdomains (11, 15), we investigated whether LY6D is localized to the raft areas. To this end, U2OS cell lysates were fractionated by sucrose density-gradient centrifugation (17). As shown in Fig. 2*I* and Fig. S6*A*, LY6D was clearly detected in the raft fraction of etoposide-treated cells (fraction 2), as determined by using caveolin 1 as a raft marker. Furthermore, LY6D was barely detected even in the whole-cell lysate of untreated cells (fraction L), suggesting that LY6D was upregulated in response to DNA damage and hence localized to the raft areas. To examine the functional consequences of LY6D raft localization on vacuole formation, the cells overexpressed with LY6D were treated with methyl-β-cyclodextrin (MβCD), a cyclic oligosaccharide that disrupts the lipid rafts by depletion of membrane cholesterol (10), and vacuole formation was monitored. The MβCD treatment effectively impaired the LY6D-induced vacuole formation in parallel with a reduction in the protein levels of LY6D and caveolin 1 in the fraction 2 (Fig. 2*J* and Fig. S1*G*, S2). To further confirm the relationship between the LY6D raft localization and vacuole formation, we constructed two transmembrane versions of LY6D, LY6D–integrinα4 and LY6D–integrinβ1, where the C-terminal region including the GPI-attachment site (98-128 aa) was replaced with the transmembrane domain of integrinα4 and integrinβ1, respectively. Integrinα4 and integrinβ1 were chosen on the basis of their nonraft localization (18). The plasma membrane localization of LY6D–integrinα4 and LY6D–integrinβ1 was verified by immunostaining assay (Fig. 2*K*). Most importantly, overexpression of integrinα4 and integrinβ1 failed to induce vacuole formation (Fig. 2*L*). These results suggest that the cell-surface localization of LY6D, more particularly in the raft areas, contributes to vacuole formation.

### LY6D-induced vacuoles are derived from macropinocytosis but not from autophagy

**Figure 3:**
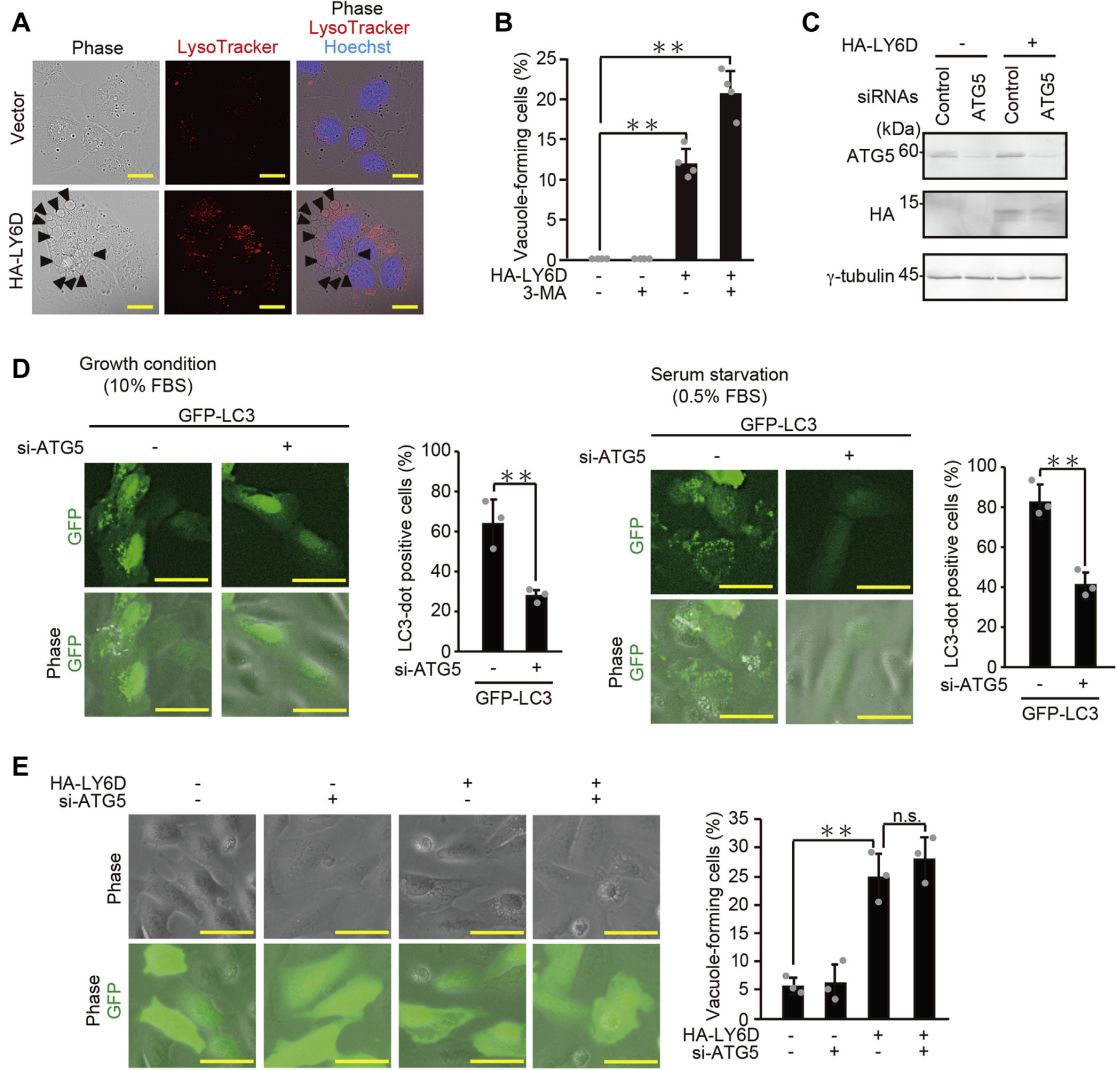
**LY6D-induced vacuoles are not derived from autophagy.***A*, U2OS cells transfected with pcDNA3-HA-LY6D were incubated with 50-nM LysoTracker for 2 h and observed under fluorescence microscope. *Arrowheads* indicate examples of cytoplasmic vacuoles. Bars, 20 μm. *B*, U2OS cells pretreated with 10-μM 3-MA for 6 h were then transfected with pcDNA3-HA-LY6D and subjected to quantification of vacuole-forming cells. *C*, U2OS cells transfected with siRNA for *ATG5* and with pcDNA3-HA-LY6D were subjected to immunoblot analysis. *D*, U2OS cells transfected with siRNA for *ATG5* and with pBABEpuro GFP-LC3 as an autophagy marker, cultured in either the growth medium (10% FBS) or serum starvation medium (0.5% FBS) for 18 h, and subjected to quantification of LC3-dot–positive cells. Representative microscopic images (*left*) and the percentage of LC3-dot–positive cells (*right*) are shown. *Bars*, 50 μm. *E*, U2OS cells transfected as in panel *C* were subjected to quantification of vacuole-forming cells. Representative microscopic images (*left*) and the percentage of vacuole-forming cells (*right*) are shown. *Bars*, 50 μm. Data are mean ± SD (*n* = 3 independent cultures except for that in panel *B*, where *n* = 4). Statistical significance is shown using the Student’s t-test analysis; ∗∗*p* < 0.01; n.s., not significant (*p* > 0.05). LY6D, lymphocyte antigen 6 complex, locus D; 3-MA, 3-methyladenine; FBS, fetal bovine serum.

We next tried to determine the origin of LY6D-induced vacuoles. Given the significant association between senescence and autophagy, a catabolic process that consists of the isolation of cytoplasmic proteins and organelles by double-membrane cytoplasmic vacuoles (autophagosomes) and their degradation through fusion with lysosomes (forming autolysosomes) (2), we first hypothesized that LY6D-induced vacuoles are derived from autophagy. To test this hypothesis, the formation of autolysosomes in the LY6D-overexpressing cells was assessed using LysoTracker, a fluorescent dye detecting low pH. Although the lysosomal compartment was more prominent in LY6D-overexpressing cells, no overlapping of the LY6D-induced vacuoles with LysoTracker signal was observed (Fig. 3, *A*). Moreover, treatment with 3-methyladenine (3-MA), an inhibitor of class III PI3K widely used to inhibit autophagy, failed to suppress the LY6D-induced vacuole formation (Fig. 3*B*). To further confirm this, we tested the effect of knockdown of *ATG5*, a key autophagy gene, on the vacuole formation. The ATG5 expression was efficiently suppressed by the transfection of siRNA (Fig. 3*C*). Furthermore, when GFP-LC3 was introduced as an autophagy marker, knockdown of *ATG5* inhibited GFP-LC3 puncta formation under normal growth conditions and serum starvation, indicating the successful suppression of autophagy by *ATG5* knockdown (Fig. 3*D*). Most importantly, depletion of *ATG5* failed to inhibit the LY6D-induced vacuole formation (Fig. 3*E*). Altogether, we concluded that the LY6D-induced vacuoles were not derived from autophagy.

**Figure 4:**
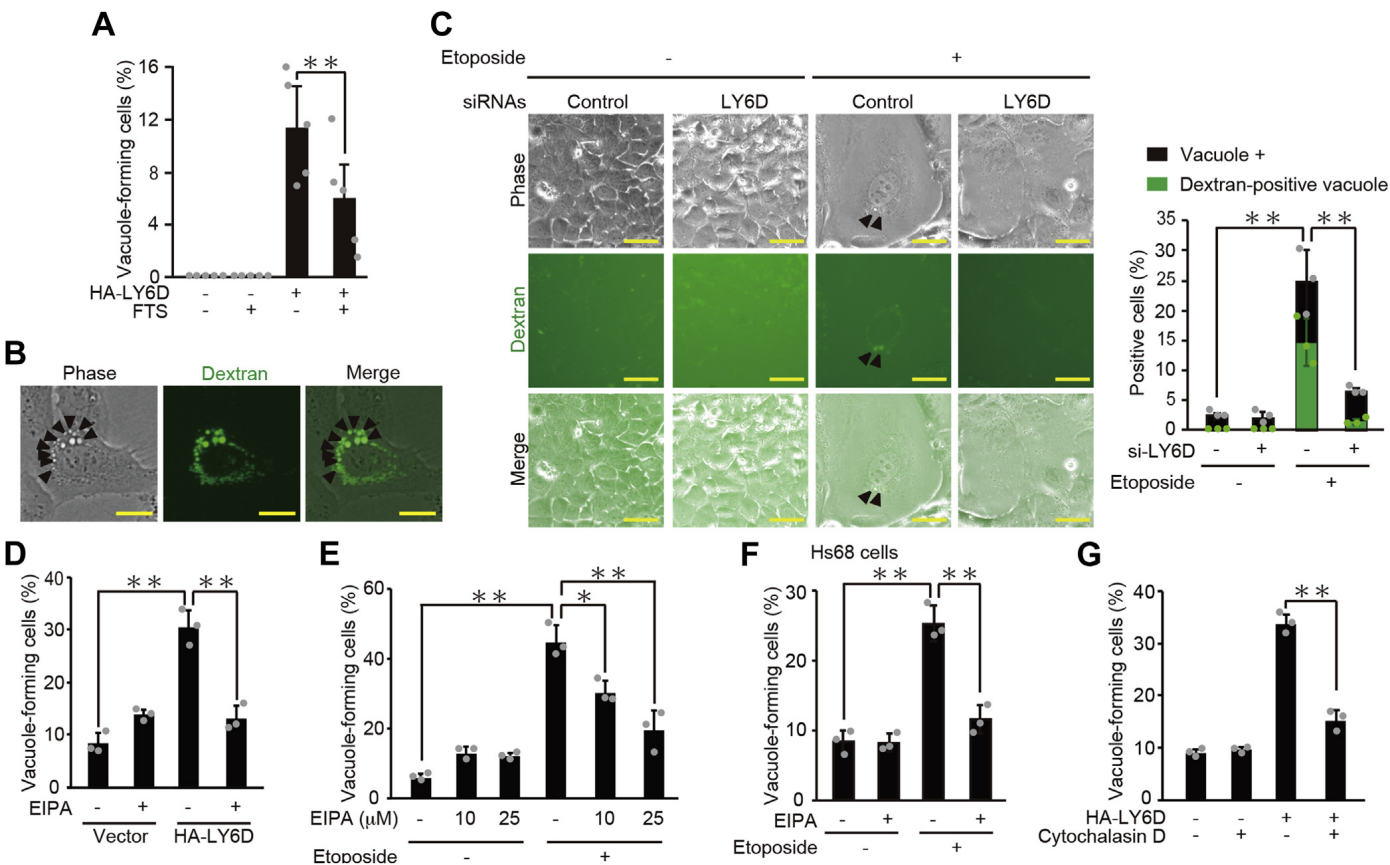
**LY6D-induced vacuoles are derived from macropinocytosis.***A*, U2OS cells pretreated with 100-μM FTS for 2 h were then transfected with pcDNA3-HA-LY6D and subjected to quantification of vacuole-forming cells. *B*, U2OS cells overexpressed with HA-LY6D were incubated with dextran-Alexa Fluor 488 (10,000 MW) for 16 h and observed under fluorescence microscope. *Arrowheads* indicate examples of cytoplasmic vacuoles. *Bars*, 20 μm. *C*, U2OS cells were transfected with *LY6D* siRNA and treated with 2-μM etoposide. After 7-day treatment, the cells were incubated with dextran-Alexa Fluor 488 (10,000 MW) for 16 h and observed under fluorescence microscope. Representative microscopic images (*left*) and the percentages of vacuole-forming cells (*black bars*) and of cells with dextran-positive vacuoles (*green bars*) (*right*) are shown. *Arrowheads* indicate the colocalization of cytoplasmic vacuoles and fluorescent dextran. *Bars*, 50 μm. *D*, U2OS cells were transfected with pcDNA3-HA-LY6D, treated with 25-μM EIPA for 16 h, and subjected to quantification of vacuole-forming cells. *E*, U2OS cells were treated with 2-μM etoposide and EIPA at the indicated concentrations for 7 days and subjected to quantification of vacuole-forming cells. *F*, Hs68 cells were treated with 0.5-μM etoposide and 10-μM EIPA for 7 days and subjected to quantification of vacuole-forming cells. *G*, U2OS cells were transfected with pcDNA3-HA-LY6D, treated with 200-nM cytochalasin D for 16 h, and subjected to quantification of vacuole-forming cells. Data are mean ± SD (*n* = 3 independent cultures except for that in panel *A*, where *n* = 5). Statistical significance is shown using the Student’s *t*-test analysis; ∗*p* < 0.05; ∗∗*p* < 0.01. LY6D, lymphocyte antigen 6 complex, locus D; EIPA, ethylisopropylamiloride; FTS, farnesyl thiosalicylic acid.

It has been reported that oncogenic Ras stimulates cytoplasmic vacuole formation (19) and that the Ras-induced vacuoles are derived from macropinocytosis (20). Therefore, to determine whether LY6D activates the Ras-mediated macropinocytic pathway, we tested the effect of farnesyl thiosalicylic acid (FTS), a Ras inhibitor, on the LY6D-induced vacuole formation. FTS effectively inhibited the vacuole formation induced by LY6D overexpression (Fig. 4*A*), raising the possibility that LY6D-induced vacuoles are derived from Ras-mediated macropinocytosis. To confirm this possibility, we incubated the LY6D-overexpressing cells with high-molecular-mass dextran, a fluid-phase macropinocytic marker (16). We observed that the LY6D-induced vacuoles were colocalized with internalized dextran (Fig. 4*B*). When combined with immunostaining for HA-LY6D, the signals of dextran and LY6D were barely overlapping with each other, suggesting that LY6D appears not to be internalized together with the newly formed vacuoles (Fig. S3). In addition, the senescence-associated vacuoles induced by etoposide also incorporated the dextran probe, which was abrogate by LY6D knockdown (Fig. 4*C*). For further confirmation, we tested the effect of ethylisopropylamiloride (EIPA), a potent inhibitor of Na^+^/H^+^ exchangers widely used to block macropinocytosis (21), on the LY6D-induced vacuole formation. The EIPA treatment effectively reduced the vacuole formation induced by LY6D (Fig. 4*D*). Moreover, etoposide-induced vacuole formation was also diminished by EIPA in both U2OS and Hs68 cells (Fig. 4, *E* and *F*). Finally, cytochalasin D, a potent inhibitor of actin polymerization required for macropinocytosis (22), abolished the LY6D-induced vacuolization (Fig. 4*G*), collectively indicating that the LY6D-induced vacuole formation was mediated by macropinocytosis.

### LY6D associates with SFK and Ras in lipid rafts of senescent cells

**Figure 5:**
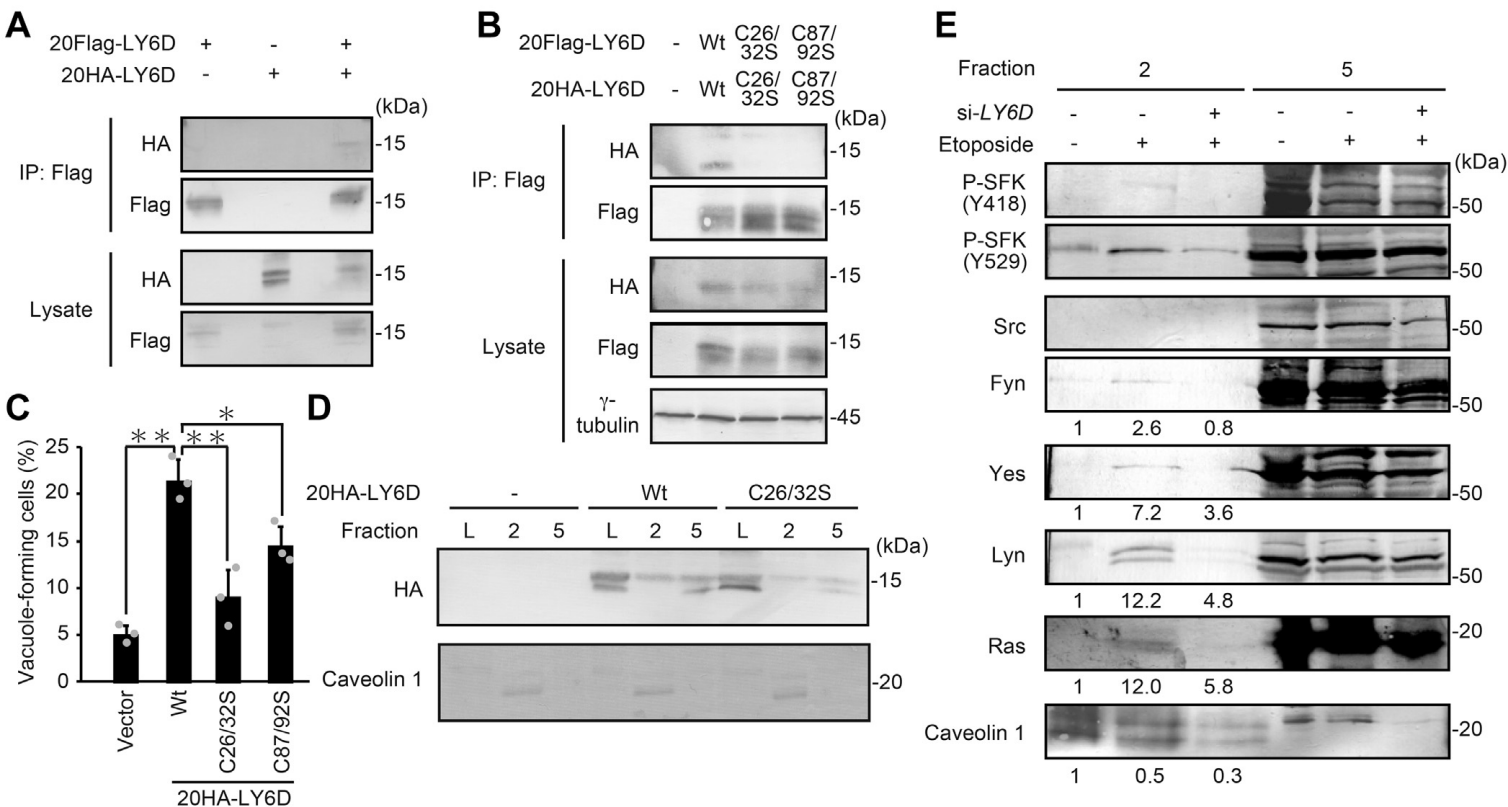
**LY6D activates Ras within cell membrane rafts mediated through particular Src family kinases**. *A*, U2OS cells transfected with pcDNA3-20Flag-LY6D and/or pcDNA3-20HA-LY6D as indicated were subjected to immunoprecipitation with anti-Flag antibody and subsequently to immunoblot analysis. *B*, U2OS cells transfected with pcDNA3-20Flag-LY6D and pcDNA3-20HA-LY6D harboring the C26/32S or C87/92S mutations as indicated were subjected to immunoprecipitation with anti-Flag antibody and subsequently to immunoblot analysis. *C*, U2OS cells cotransfected with pcDNA3-20HA-LY6D harboring the C26/32S or C87/92S mutations and pEGFP as a transfection marker for 24 h were observed under microscope. The percentage of vacuole-forming cells in GFP-expressing cells is shown. Data are mean ± S.D. (*n* = 3 independent cultures). Statistical significance is shown using the Student’s t-test analysis; ∗*p* < 0.05, ∗∗*p* < 0.01. *D*, Lysates of U2OS cells overexpressed with 20HA-LY6D-Wt or C26/32S were fractionated by sucrose density-gradient centrifugation, and the raft-containing fraction (fraction 2) and the nonraft fraction (fraction 5) were subjected to immunoblot analysis. Caveolin 1 was used as a raft marker, indicating that fraction 2 contained the raft fraction. Fraction L represents whole-cell lysates. The results of different batch experiments are shown in Fig. S6*B*. *E*, lysates of LY6D-depleted U2OS cells treated with etoposide were fractionated by sucrose density-gradient centrifugation, and the raft-containing fraction (fraction 2) and the nonraft fraction (fraction 5) were subjected to immunoblot analysis. Caveolin 1 was used as a raft marker and used for normalization of raft recovery. The relative protein levels of Fyn, Yes, Lyn, and Ras in fraction 2 normalized to the caveolin 1 level were determined using NIH ImageJ software and indicated at the *bottom* of each lane. The results of different batch experiments are shown in Fig. S6*C*. LY6D, lymphocyte antigen 6 complex, locus D.

We next sought to delineate the molecular mechanism(s) underlying the LY6D-induced vacuole formation. Because several GPI-anchored proteins are known to form homomultimers or heteromultimers at the raft areas (11, 23), we tested whether the LY6D proteins interact with each other in the cells by coimmunoprecipitation assay. To this end, we constructed LY6D containing an internal FLAG tag (20Flag-LY6D) or HA tag (20HA-LY6D) between amino acid residues 20 and 21 because the N-terminal signal sequence of LY6D (residues 1–20) is cleaved upon entry of the protein into endoplasmic reticulum, and thus N-terminal tags cannot be captured by tag-specific antibodies. The 20Flag-LY6D and 20HA-LY6D were coexpressed in U2OS cells, and 20Flag-LY6D was immunoprecipitated with anti-Flag antibody (Fig. 5*A*). As a result, only when both of the tagged LY6D overexpressed, the coprecipitated 20HA-LY6D was evidently detected by immunoblot analysis, suggesting that LY6D forms multimers in the cells like other GPI-anchor proteins. LY6D has a well-conserved LY6/uPAR domain that is characterized by five disulfide bonds and three flexible loops extended from the cysteine-rich core. This three-fingered structural motif is typically involved in the protein–protein interaction, although the binding partners of LY6D are currently unidentified. To elucidate whether the formation of LY6D multimers leads to the vacuole formation or not, we generated two LY6D mutants in which the second disulfide bond (Cys26-Cys32) and fifth disulfide bond (Cys87-Cys92) were disrupted by replacing Cys26/32 and Cys87/92 with serine (C26/32S and C87/92S), respectively, because the disruption of the second or fifth disulfide bonds in GPIHBP1, a member of the LY6 family, was shown to effectively inhibit the binding of GPIHBP1 to its binding partner, lipoprotein lipase (24, 25). Immunoprecipitation analysis revealed that multimer formation of LY6D was inhibited in the C26/32S and C87/92S mutants (Fig. 5*B*), suggesting that these disulfide bonds (Cys26-Cys32 and Cys87-Cys92) play a vital role in the LY6D multimerization. Furthermore, vacuole formation was hardly induced by overexpression of the C26/32S mutant, whereas overexpression of the C87/92S mutant resulted in a slight decrease in the percentage of vacuole-forming cells than WT (Fig. 5*C*). Moreover, the C26/32S mutation reduced the raft localization of LY6D (Fig. 5*D and* Fig. S6*B*). These results suggest that the multimerization of LY6D is required for the induction of vacuole formation.

Because it has been shown that the clusters of GPI-anchored proteins can act as signaling platforms for intracellular signaling proteins such as SFK (11, 12, 13, 14), we investigated whether or not intracellular signaling proteins, such as SFK and Ras, are accumulated in the LY6D-contained raft fraction (Fig. S4). The sucrose density-gradient centrifugation revealed that Ras was slightly accumulated in the raft fraction (fraction 2) when LY6D was overexpressed. Furthermore, the bands ranging from 50 to 60 kDa were detected with an antibody against phosphorylated tyrosine in the LY6D-overexpressing cells, leading us to hypothesize that SFK accumulate in the lipid rafts upon LY6D expression because SFK have molecular weights in the range of 50 to 60 kDa. In fact, phosphorylated SFK (Y418) in the raft fraction was slightly increased by LY6D overexpression as assessed with anti-phosphorylated SFK (Y418) antibody. To further explore this, immunoblot analysis was performed using a series of antibodies against the members of SFK (Src, Yes, Fyn, Fgr, Lyn, Hck, and Blk) (Fig. S4). As a result, among the SFK family members, Fyn and Lyn were found to be accumulated in the raft of LY6D-overexpressing cells. In addition, the etoposide treatment induced the recruitment of Fyn, Lyn, and Ras to the raft, all of which were abolished by depletion of LY6D (Fig. 5*E* and Fig. S6*C*). These results suggest that a cluster of signaling proteins including SFK and Ras is formed in the lipid raft during senescence in an LY6D-dependent manner.

**Figure 6:**
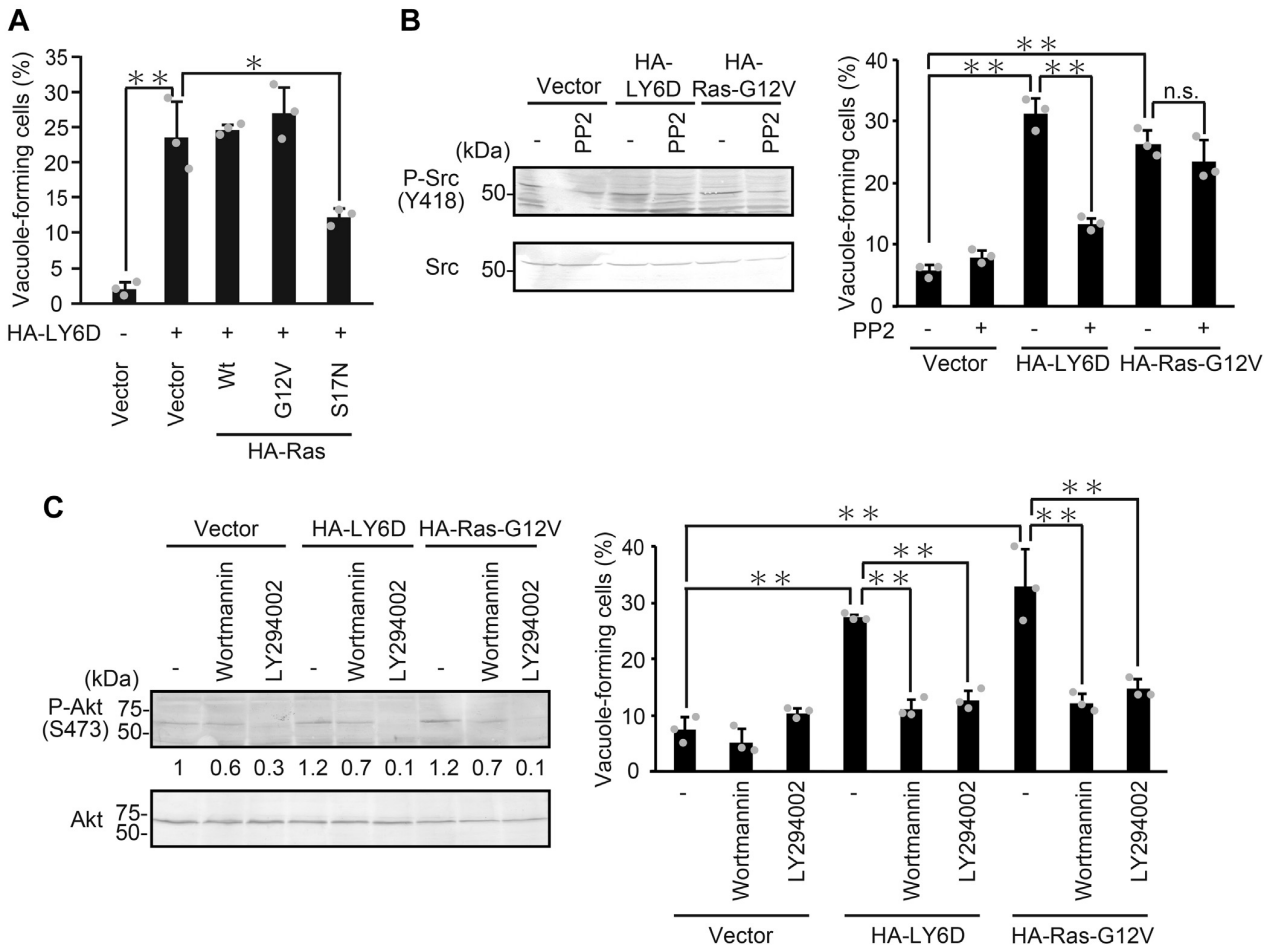
**LY6D induces vacuole formation through the Ras-PI3K pathway**. *A*, U2OS cells transfected with pcDNA3-HA-LY6D in combination with pcDNA3-HA containing Wt-Ras, G12V-Ras, and S17N-Ras as indicated were subjected to quantification of vacuole-forming cells. *B*, U2OS cells transfected with pcDNA3-HA containing LY6D and Ras-G12V and treated with 10-μM PP2 for 16 h were subjected to immunoblot analysis (*left panel*) and quantification of vacuole-forming cells (*right panel*). *C*, U2OS cells transfected with pcDNA3-HA containing LY6D and Ras-G12V were treated with 1-μM wortmannin and 20-μM LY294002 as indicated for 16 h and subjected to immunoblot analysis (*left panel*) and quantification of vacuole-forming cells (*right panel*). The Akt phosphorylation levels relative to the total Akt level were quantified using NIH ImageJ software and indicated at the *bottom* of each lane. Data are mean ± S.D. (*n* = 3 independent cultures). Statistical significance is shown using the Student’s *t*-test analysis; ∗*p* < 0.05; ∗∗*p* < 0.01; n.s., not significant (*p* > 0.05). LY6D, lymphocyte antigen 6 complex, locus D.

Next, to investigate the functional relationships between the accumulation of signaling factors and macropinocytosis, we tested the impact of various Ras mutants on macropinocytosis (26, 27). Overexpression of WT-Ras or constitutive active form of Ras (G12V), but not the dominant-negative Ras (S17N), evidently induced vacuole formation (Fig. S5*A*), and the Ras-induced vacuole formation was remarkably inhibited by the treatment with FTS (Fig. S5*B*), all of which indicates that macropinocytosis induced by Ras is dependent on Ras activity itself as previously reported (20). To examine the functional relevance between LY6D and Ras, U2OS cells were co-overexpressed with LY6D and each of the Ras mutants, and the vacuole formation was monitored (Fig. 6, *A*). As a result, both Wt-Ras and active G12V-Ras did not influence the LY6D-induced macropinocytosis, but dominant-negative S17N-Ras impaired it, suggesting that LY6D and Ras function in the same signaling pathway and Ras acts downstream of LY6D to induce macropinocytosis. Moreover, PP2, a specific SFK inhibitor, suppressed the LY6D-induced macropinocytosis but not the Ras-induced one (Fig. 6*B*). These results collectively indicate that the LY6D-SFK-Ras axis functions to induce macropinocytosis.

We next set out to elucidate the signaling pathway that acts downstream of LY6D-SFK-Ras to induce macropinocytosis. Ras can activate several different downstream pathways such as MAP kinase, Ral, and PI3K pathways (28, 29). We found that the treatment with U0126, a MAPKK inhibitor, did not suppress the LY6D-induced macropinocytosis (Fig. S5*C*), suggesting that the MAP kinase pathway is dispensable for the induction of macropinocytosis. Therefore, to determine which pathway(s) is responsible for the LY6D-induced vacuole formation, we constructed three Ras mutants, T35S and E37G, and Y40C, that only interact with each of the effectors, Raf (MAP kinase), Ral-GEF (Ral), and PI3K (PI3K pathway), respectively (28), and tested their effect on macropinocytosis. As a result, overexpression of the Y40C mutant, but not the T35S and E37G mutants, effectively induced vacuole formation (Fig. S5*D*), which raises the possibility that the PI3K pathway induced macropinocytosis. Consistent with the previous report that the Y40C mutant interacts with and activates PI3K, immunoblot analysis revealed that overexpression of the Y40C mutant activated the PI3K pathway, as judged by an increase in the phosphorylation level of AKT, a downstream effector of PI3K (Fig. S5*D*, *left panel*). In addition, class I PI3K inhibition by inhibitors, Wortmannin and LY294002, effectively suppressed both LY6D-induced and Ras-induced macropinocytosis (Fig. 6*C*). These results suggest that the LY6D-SFK-Ras axis induces macropinocytosis through the activation of the PI3K pathway.

### LY6D-induced macropinocytosis contributes to senescent cell survival through incorporation of extracellular nutrient

**Figure 7:**
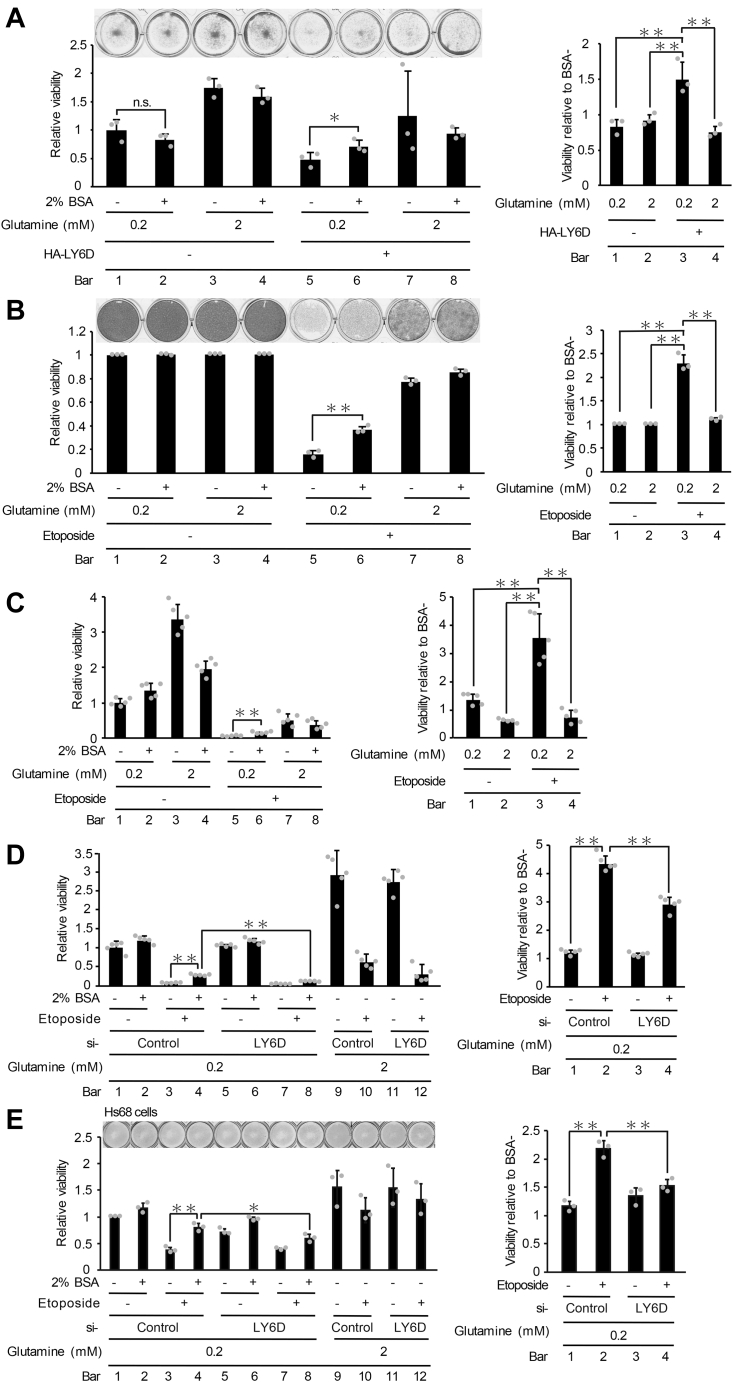
**LY6D-induced macropinocytosis contributes to senescent cell survival through incorporation of extracellular nutrients**. *A*, U2OS cells transfected with pcDNA3-HA-LY6D were then cultured in a low-glutamine medium (containing 0.2-mM glutamine) or in a normal medium (containing 2-mM glutamine) with/without 2% BSA as indicated for 7 days. The relative cell viability was determined by crystal violet staining. The *left* panel shows cell viability relative to the negative control cells (bar 1), and the *right* panel shows viability rates of cells with to without BSA supplementation (e.g. the ratio of bar 2/bar 1, bar 4/bar 3, and so on). *B* and *C*, U2OS cells treated with 2-μM etoposide for 7 days were then cultured in a low-glutamine medium or in a normal medium with/without 2% BSA as indicated for 7 days. The relative cell viability was determined by crystal violet staining (*B*) and the WST-1 cell-viability assay (*C*). The *left* panels show cell viability relative to the negative control cells (bar 1), and the right panels show viability rates of cells with to without BSA supplementation as described in panel *A*. *D* and *E*, *LY6D*-depleted U2OS (*D*) and Hs68 (*E*) cells were treated as in panels *B* and *C*, and the relative cell viability was determined by the WST-1 assay (*D*) and crystal violet staining (*E*). The *left* panels show cell viability relative to the negative control cells (bar 1), and the *right* panel shows viability rates of cells with to without BSA supplementation as described in panel *A*. Data are mean ± SD (*n* = 3 independent cultures except for those in panels *C* and *D*, where *n* = 5). Statistical significance is shown using the Student’s *t*-test analysis; ∗*p* < 0.05; ∗∗*p* < 0.01. LY6D, lymphocyte antigen 6 complex, locus D; BSA, bovine serum albumin.

We next aimed to determine the physiological significance of senescence-associated induction of macropinocytosis. Macropinocytosis has been recently reported to function as an amino acid supply route in Ras-transformed cancer cells (15, 16). Commisso *et al* have shown that a glutamine deprivation–induced decline in survival of Ras-transformed cells is recovered by extracellular supplementation with bovine serum albumin (BSA) in a macropinocytosis-dependent manner, leading to the conclusion that Ras-induced macropinocytosis contributes to cancer cell survival through the incorporation of extracellular fluid (16). Given the similarity in the high energy demand between cancer and senescent cells (2), it is possible that macropinocytosis-mediated incorporation of extracellular fluid can enhance survival of senescent cells as is the case in cancer cells. We therefore tested whether LY6D-induced macropinocytosis can promote survival of senescent cells (Fig. 7). Cell viability was measured by crystal violet staining after 7 days of culture in a low-glutamine medium (0.2 mM) or in a normal medium (containing 2-mM glutamine) with/without 2% BSA (Fig. 7*A*). We found that cell viability was remarkably decreased when cultured in the low-glutamine medium (compare bars 1 with 3 in Fig. 7*A*, *left panel*) as previously reported (16). More importantly, the low-glutamine–induced decline in cell survival was partly recovered by the combination of the BSA supplementation and LY6D overexpression (compare bars 5 with 6 in Fig. 7*A*, *left panel*) but not by either BSA or LY6D alone (compare bars 1 with 2, or 1 with 5 in Fig. 7*A*, *left panel*). To clearly show the effects of BSA supplementation on cell survival, the data of Fig. 7*A*, *left panel* were re-represented in Fig. 7*A*, *right panel*, in which viability rates of cells with to without BSA supplementation at each condition are shown. These results support the hypothesis that LY6D-induced macropinocytosis contributes to cell survival through the uptake of the extracellular fluid. To expand this observation in nonsenescent cells into the senescence context, we examined whether the BSA supplementation restores cell viability in etoposide-induced senescent cells (Fig. 7*B*). U2OS cells were treated with etoposide for 7 days and subsequently cultured in the high- or low-glutamine medium with/without BSA for additional 7 days. Nonsenescent cells were not sensitive to the low-glutamine treatment, whereas viability of senescent cells markedly decreased by glutamine deprivation (compare bars 5 with 7 in Fig. 7*B*, *left panel*), which was partially restored by the BSA supplementation (compare bars 6 with 5 in Fig. 7*B*, *left panel*, and see bar 3 in Fig. 7*B*,*right panel*). Because the BSA addition by itself did not improve viability of senescent cells under the high glutamine condition, these results suggest that senescence-associated macropinocytosis promote senescent cell survival through the incorporation of extracellular BSA. To confirm this using a biochemical approach, cell viability was measured by another method, the WST-1 assay, in which cell viability is monitored by the activity of mitochondrial dehydrogenases (Fig. 7*C*). Consistent with the results obtained from crystal violet staining, the WST-1 assay showed that the low glutamine–induced decline in survival of senescent cells was recovered by the BSA supplementation (compare bars 6 with 5 in Fig. 7*C* left panel, and see bar 3 in Fig. 7*C*, *right panel*). Furthermore, to determine whether the effect of BSA supplementation on improvement in senescent cell survival is dependent on LY6D, the BSA-mediated recovery of viability was compared between LY6D-depleted and nondepleted cells (Fig. 7*D*). siRNA-mediated knockdown of LY6D clearly impaired the survival improvement by the BSA addition (compare bars 8 with 4 in Fig. 7*D*, *left panel*, and compare bars 4 with 2 in Fig. 7*D*, *right panel*). Finally, the same experiment was performed using Hs68 cells (Fig. 7*E*). Senescent Hs68 cells were more susceptible to the low-glutamine condition than nonsenescent cells (compare bars 3 with 1 in Fig. 7*E*, *left panel*), which was mitigated by the BSA supplementation as is the case in U2OS cells (compare bars 3 with 4 in Fig. 7*E*, *left panel*). In addition, the BSA-mediated recovery of senescent cell viability was again dependent on LY6D (compare bars 8 with 4 in Fig. 7*E*, *left panel*, and compare bars 4 with 2 in Fig. 7*E*, *right panel*). These results collectively indicate that senescence-associated macropinocytosis induced by LY6D contributes to survival of senescent cells in a certain low-nutritional status.

## Discussion

Although senescent cells acquire various phenotypic changes such as enlarged and flattened cell morphology, increased granularity, and vacuole formation, the molecular mechanisms underlying each phenotype are currently unclear (1,2). We have previously found that LY6D is upregulated in both replicative and premature senescent cells, suggesting the general physiological roles of LY6D in the senescence program (6). LY6D is an extracellular GPI-anchored protein attached to the outer surface of the plasma membrane, whose physiological function still remains ambiguous (5). In the present study, we provided evidence that LY6D played a causative role in the senescence-associated vacuole formation of tumor and normal cells. Furthermore, the LY6D-induced vacuole formation occurred through the induction of Ras-dependent macropinocytosis, a distinct form of endocytosis. Unlike other major endocytic pathways, macropinocytosis is independent of clathrin and dynamin and characterized by the formation of large irregular cytoplasmic vacuoles, so-called macropinosomes (15). In general, the macropinocytic pathway is considered to be initiated by binding of growth factors to receptor tyrosine kinases, which in turn activates intracellular signaling factors such as SFK, Ras, and PI3K, leading to actin polymerization. However, we observed that the highly expressed LY6D spontaneously formed multimers, which resulted in the macropinocytic vacuole formation independent of growth factor stimulation. These results suggest that macropinocytosis can be initiated by signals other than the growth factor stimulation, in this case, by the upregulation and subsequent multimerization of GPI-anchored protein.

Furthermore, we revealed by sucrose density-gradient centrifugation that LY6D was accumulated in the lipid raft areas in response to DNA damage. The lipid rafts are platforms for concentrating signaling proteins, such as SFK and Ras, and can influence signal transduction (9, 10, 30). Mounting evidence indicates that GPI-anchored proteins are accumulated in the rafts to modulate the intracellular signaling (11, 15, 31). In particular, urokinase-type plasminogen activator receptor (uPAR), a GPI-anchored membrane receptor, forms dimers preferentially in the rafts, which enhances the interaction between uPAR and its binding partner, vitronectin (23). Considering that uPAR and LY6D share the LY6/uPAR domain, a highly conserved structural domain involved in protein–protein interaction (5), it is possible that the LY6D multimerization alters its affinity to functional binding partner(s) and thus provides signaling cues to activate downstream signaling factors, thereby inducing macropinocytosis. If this is the case, SFK and Ras are the most promising candidates for transducing the macropinocytosis signal from LY6D to the terminal actin polymerization machinery because SFK and Ras were observed to be accumulated in the rafts upon senescence in an LY6D-dependent manner. In addition, we found that the inhibition of Ras effectively impaired LY6D-induced macropinocytosis, and the SFK inhibition suppressed LY6D-induced macropinocytosis but not Ras-induced one, indicating that SFK lies between LY6D and Ras, that is, LY6D induces macropinocytosis through the SFK-Ras signaling cascade. In support of our findings, SFK have been reported to be activated by the accumulation of GPI-anchored proteins in the raft areas (12, 13, 14), and at the same time, both SFK and Ras are known to be capable of activating the macropinocytic pathway (19, 20, 32). Although so far there are no reports of the role of GPI-anchored proteins in the direct activation of Ras, SFK are well known to activate Ras through the conventional Shc-Grb2-SOS pathway (33).

How the SFK-Ras cascade induces macropinocytosis? Ras can activate multiple downstream pathways, including MAP kinase, Ral, and PI3K pathways (28, 29). Among these pathways, we identified PI3K as the most probable effector for the induction of macropinocytosis, by using three Ras mutants that specifically activates each of the aforementioned three pathways. Furthermore, the inhibition of PI3K prominently suppressed both LY6D-induced and Ras-induced macropinocytosis. Consistent with these results, PI3K has been reported to contribute to macropinocytosis (34). PI3K is activated by Ras through its Ras-binding domain and produces the membrane lipid PIP3. PIP3 can recruit downstream effector molecules such as Akt and a set of myosin-I motor proteins that have important roles in shaping macropinosomes. Our results, in conjunction with previous reports by others, suggest that the LY6D-induced Ras activation leads to the PI3K activation and finally to the induction of macropinocytosis.

Despite the loss of proliferative ability, senescent cells are known to have a high energy requirement (2). Actually, senescent cells typically display an enlarged morphology, contain more proteins per cell than proliferating cells, and maintain an active synthesis of proteins. A recent study has reported that Ras-transformed cancer cells internalize and subsequently degrade extracellular proteins through macropinocytosis to support their high metabolic rate (16). Given the similarity between cancer and senescent cells in terms of the high metabolic activity, it is possible that LY6D-induced macropinocytosis contributes to meet the increased energy demand of senescent cells. In support of this possibility, we revealed that the extracellular supplementation of BSA restored the glutamine deprivation–induced decline in senescent cell survival in an LY6D-dependent manner. Recently, several studies have shown the correlation between LY6D expression level and poor patient outcomes in multiple cancer types such as breast, lung, gastric, ovarian, and prostate and other carcinomas (35). Considering the aforementioned report on macropinocytosis-mediated cancer cell survival, it is possible that the LY6D-mediated macropinocytosis can promote cell survival not only in senescent but also in cancer cells. Because the functional relevance of LY6D to tumorigenesis or tumor maintenance has yet to be described, our study hopefully leads to a better understanding of the molecular mechanism underlying malignant progression of cancer cells. Therefore, whether or not LY6D promotes the senescent cell survival *in vivo* and whether or not LY6D induces macropinocytosis in malignant cancer cells are the key questions for future research. Regardless of their outcomes, our results clearly show that LY6D is responsible for the senescence-associated vacuole formation through the induction of Ras-dependent macropinocytosis, which may shed new light on the LY6D roles in the survival of senescent cells.

## Experimental procedures

### Cell culture, treatment, and transfection

U2OS (a human osteosarcoma line; ATCC, Rockville, MD) and Hs68 (normal human diploid fibroblasts; IFO50350, JCRB Cell Bank) (36) cells were cultured in Dulbecco's modified Eagle's medium (Wako, Osaka, Japan) supplemented with 10% fetal bovine serum. The cells were treated with etoposide (Sigma Aldrich, St Louis, MO) or bleomycin (Wako) to induce DNA double-strand breaks. For senescence induction, U2OS and Hs68 cells were treated with 2- and 0.5-μM etoposide, respectively, or 2-μM bleomycin for 48 h and cultured in the medium without the drugs for additional 5 days to develop senescent phenotypes (6, 7, 8, 37). Transfection with expression vectors was carried out using Effectene Transfection Reagent (Qiagen, Venlo, Netherlands) according to the manufacturer’s instruction. MβCD (Sigma Aldrich) was used to disrupt raft fraction. EIPA (Cayman chemical, Ann Arbor, MI) was used to inhibit macropinocytosis (21). For lysosome staining, the cells were incubated with 50-nM LysoTracker Red DND-99 (Life Technologies, Carlsbad, CA) for 2 h before observation. 3-MA (Merck Millipore, Darmstadt, Germany) or FTS (Merck Millipore) was added to the medium 3 and 6 h before transfection, respectively. Dextran Alexa Fluor 488 10,000 MW (Life Technologies) was used to study macropinocytosis. U0126 (Wako) was used to inhibit MEK; wortmannin and LY294002 (both obtained from Merck Millipore) were used to inhibit PI3K. To disrupt actin polymerization, cytochalasin D (Merck Millipore) was used. For some conditions, media was supplemented with BSA (fraction V, fatty-acid–free, nuclease-free, and protease-free, Merck Millipore). Stock solutions of etoposide, FTS, U-0126, wortmannin, LY294002, and cytochalasin D were prepared in dimethyl sulfoxide, whereas bleomycin, MβCD, EIPA, and 3-MA were dissolved in water.

### Plasmid constructions

For the construction of pcDNA3-HA-LY6D-Wt, an expression vector for N-terminal hemagglutinin (HA)-tagged human full-length (1-387 bp) LY6D, the LY6D cDNA was amplified with a pair of primers (a forward primer: 5’-CGTGCTCGGAATTCATGAGGACAGCATTGCTGCT-3’ and a reverse primer: 5’-CGTGCTCGGCGGCCGCTCACAGGCTGGGGGCTAAGA-3’) using a cDNA sample prepared from U2OS cells as a template. The resultant fragments were digested with Eco RI and Not I and cloned into pcDNA3 vector (Invitrogen, Carlsbad, CA). The fragment of signal sequence–deleted LY6D (Δ1-20 LY6D) was amplified with the same reverse primer and a forward primer 5’-GCTGCTTCGAATTCCTGCGCTGCCACGTGTGCA-3’ and cloned into the pcDNA3 to generate pcDNA3-HA-LY6D-Δ1-20. For the construction of LY6D containing an internal HA tag (20HA-LY6D) or Flag tag (20Flag-LY6D) between amino acid residues 20 and 21, DNA fragments corresponding to the 5’ of LY6D (1-60 bp), the HA (TACCCATACGACGTGCCAGACTACGCC) or Flag tag (GACTACAAAGACGATGACGACAAG) sequence, and the 3’ of LY6D (61-387 bp) were ligated in this order to the pcDNA3 vector by using the In-Fusion cloning system (Takara Bio, Shiga, Japan) to generate pcDNA3-20HA-LY6D and pcDNA3-20Flag-LY6D. For the construction of transmembrane domain–containing versions of LY6D, DNA fragments corresponding to the N-terminal half of 20HA-LY6D (1-318 bp) and the transmembrane domain of human integrinα4 (2932-3096 bp) or integrinβ1 (2185-2394 aa) were ligated to the pcDNA3 vector by using the In-Fusion cloning system to construct pcDNA3-20HA-LY6D-integrinα4 and pcDNA3-20HA-LY6D-integrinβ1, respectively. The plasmid encoding GFP-fused human LC3, pBABEpuro GFP-LC3 (plasmid #22405), was obtained from Addgene (Cambridge, MA). For the generation of HA- and Flag-tagged C26/32S-LY6D and C87/92S-LY6D mutants, PCRs were performed using mutagenic primers (a forward primer: TGCGCTGCCACGTGTCAACCAGCTCCAGCAACTCAAAGCATTCTGTGGTC and a reverse primer: TGCGCTGCCACGTGTCAACCAGCTCCAGCAACTCAAAGCATTCTGTGGTC for C26/32S-LY6D and a forward primer: GCTCCACCCAGTGCTCACAGGAGGACCTGTCAAATGAGAAGCTGCAC and a reverse primer: GTGCAGCTTCTCATTTGACAGGTCCTCCTGTGAGCACTGGGTGGAGC for C87/92S-LY6D) using pcDNA3-20HA-LY6D or pcDNA3-20Flag-LY6D as the template to generate pcDNA3-20HA-LY6D-C26/32S, pcDNA3-20HA-LY6D-C87/92S, pcDNA3-20Flag-LY6D-C26/32S, and pcDNA3-20Flag-LY6D-C87/92S. To construct the expression vectors, pcDNA3-HA-Ras-Wt, pcDNA3-HA-Ras-G12V, and pcDNA3-HA-Ras-S17N, that contain full-length human H-Ras-Wt, G12V, and S17N, respectively, the corresponding cDNA fragments were amplified with a pair of primers (a forward primer: GCGAATTCATGACGGAATATAAGCTGGTG and a reverse primer: GCAGCGGCCGCTCAGGAGAGCACACACTTG for all three constructs) using pCMV-HA-Ras-Wt, pCMV-HA-Ras-G12V, and pCMV-HA-Ras-S17N as the templates (kindly provided from K. Kaibuchi, Nagoya university, Japan). The resultant fragments were digested with Eco RI and Not I and cloned into downstream of the HA tag sequence in the pcDNA3-HA vector. For the construction of H-Ras mutants (T35S and E37G, and Y40C), PCRs were performed using mutagenic primers (a forward primer: TGGACGAATACGACCCCTCTATAGAGGATTCCTACCGGA and a reverse primer: TCCGGTAGGAATCCTCTATAGAGGGGTCGTATTCGTCCA for T35S; a forward primer: ACGAATACGACCCCACTATAGGCGATTCCTACCGGAAGCAG and a reverse primer: CTGCTTCCGGTAGGAATCGCCTATAGTGGGGTCGTATTCGT for E37G; and a forward primer: CCCCACTATAGAGGATTCCTGCCGGAAGCAGGTGG and a reverse primer: CCACCTGCTTCCGGCAGGAATCCTCTATAGTGGGG for Y40C) with pCMV-G12V-Ras as the template to generate pcDNA3-HA-Ras-G12V/T35S, pcDNA3-HA-Ras-G12V/E37G, and pcDNA3-HA-Ras-G12V/Y40C.

### Antibodies

Anti-LY6D antibody (sc-373838), HRP-conjugated anti-rat antibody (sc-2032), anti-Pan P-Tyr PY99 antibody (sc-7020), anti-c-Src antibody (sc-19), anti-c-Yes antibody (sc-8403), anti-c-Fgr antibody (sc-130), anti-Lyn antibody (sc-015), anti-Hck antibody (sc-072), and anti-Blk antibody (sc-329) were obtained from Santa Cruz Biotechnology (Santa Cruz, CA); anti-ATG5 (#12994), anti-P-Erk (T202/204) antibody (#9101S), anti-Erk antibody (#9102S), anti-P-Akt (S473) antibody (#9271S), and anti-Akt antibody (#9272S) were from Cell Signaling Technology (Beverly, MA); anti–γ-tubulin antibody (T6557) and anti–FLAG M2 monoclonal antibody (F3165) were from Sigma Aldrich; anti–P-SFK (Y418) antibody (44--660G) and anti–P-SFK (Y529) antibody (44--662G) were from Thermo Fisher Scientific (Waltham, MA); anti-HA 3F10 monoclonal antibody (1867423) and anti-HA 12CA5 monoclonal antibody (11666606) were from Roche (Basel, Switzerland); HRP-conjugated anti-mouse antibody (W4021) was from Promega; anti-Ras antibody (#06-570) was from Merck Millipore; anti-Fyn antibody (PC32) was from Oncogene science (Uniondale, NY); anti-caveolin 1 antibody was from BD Biosciences (San Jose, CA).

### Immunoblot analysis

The cells were lysed in the lysis buffer (1% Nonidet P-40, 50 mM Tris-HCl [pH 7.5], 5 mM EDTA, 150 mM NaCl, 20 mM NaF, 20 mM β-glycerophosphate, 10 μg/ml leupeptin, 10 μg/ml aprotinin, 1 mM phenylmethanesulfonyl fluoride), and the lysates were separated by SDS-PAGE and blotted onto Immobilon polyvinylidene difluoride membrane (Merck Millipore). Each protein was detected using primary antibodies as indicated, HRP-conjugated secondary antibodies, and the ECL detection reagent (GE Healthcare, Buckinghamshire, England).

### Senescence assay

For detection of SA-β-Gal activity, the senescence β-galactosidase staining kit (Cell Signaling Technology) was used according to the manufacturer’s instruction. Briefly, the cells were fixed with 2% formaldehyde/0.2% glutaraldehyde for 15 min and washed with PBS. After incubation with SA-β-Gal staining solution (1 mg/ml 5-bromo-4-chloro-3-indolyl-β-D-galactoside, 40 mM citric acid/sodium phosphate [pH 6.0], 5-mM potassium ferrocyanide, 5-mM potassium ferricyanide, 150-mM NaCl, 2-mM MgCl_2_) for 24 h, the cells were examined under fluorescence microscope (model BZ-8000; Keyence, Osaka, Japan). Senescent cells were identified as blue-stained cells with phase contrast, and at least 100 cells in randomly selected microscopic fields were counted to determine the percentage of SA-β-Gal–positive cells. For EdU incorporation assay, U2OS cells were labeled with EdU for 3 h before fixation, and then, EdU incorporation was detected using the Click-iT EdU Imaging Kit (Life Technologies) according to the manufacturer's instructions. The stained cells were observed under fluorescence microscope (model BZ-9000; Keyence).

### RNA interference

ON-TARGETplus Smart Pool siRNA for LY6D (L-012615-01) and its control siRNA (D-001810-10) were from GE Healthcare Dharmacon (Lafayette, CO). ATG5 siRNA (sc-41445) was obtained from Santa Cruz Biotechnology. Cells were seeded and transfected with 30-nM siRNA using HiPerFect Transfection Reagent (Qiagen, Venlo, Netherlands) according to the manufacturer’s instruction.

### Immunofluorescence

For immunofluorescence analysis, the cells were fixed with 4% paraformaldehyde and permeabilized in 0.5% Triton X-100 and then incubated with primary antibodies in Can Get Signal immunostain Solution B (TOYOBO, Osaka, Japan) overnight at 4 °C followed by incubation with the Alexa Fluor 488–conjugated secondary antibody (Life Technologies) for 1 h at room temperature. After staining cell nuclei with Hoechst 33258, the cells were observed under fluorescence microscope (model BZ-8000; Keyence).

### Subcellular fractionation

To obtain raft fractions, cells were lysed in Triton X-100–containing lysis buffer (1% Triton X-100, 20-mM Tris-HCl [pH 7.5], 1-mM EDTA, 1-mM EGTA, 10-mM β-mercaptoethanol, 1-mM Na3VO4, 10 μg/ml leupeptin, 20-μM p-amidinophenylmethanesulfonyl fluoride, hydrochloride, 150-mM NaCl), and the lysates were collected and centrifuged at 10,000*g* for 10 min. The supernatants were mixed with equal volumes of ice-cold 85% sucrose. The resulting mixtures (1 ml) were placed in an ultracentrifuge tube. A stepwise sucrose gradient (2.5 ml of 30% and 1.5 ml of 5% sucrose) was layered over the mixtures. The samples were centrifuged at 100,000*g* for 20 h in an SW55Ti rotor (Beckman Coulter, Brea, California). After the centrifugation, 1-ml aliquots of 5 fractions were collected from the top to the bottom of the tubes. Fraction 2 was pooled as raft fraction, whereas fraction 5 was pooled as nonraft fraction (17).

### Immunoprecipitation

For immunoprecipitation experiments, cells were lysed in Triton X-100–containing lysis buffer. The lysates were incubated with 2 μg of anti-FLAG M2 monoclonal antibody together with Protein G Sepharose (GE Healthcare) for 2 h at 4 °C with constant rotation. The bound proteins were eluted by addition of the SDS sample buffer (50-mM Tris-HCl [pH 6.8], 2% SDS, 5% 2-mercaptoethanol, 0.1% bromophenol blue, 10% glycerol). The obtained cell lysates and eluates were separated by SDS-PAGE and immunoblotted with the indicated antibodies.

### Cell-viability assay

Cell viability was measured by crystal violet staining or by a cell proliferation reagent WST-1 (Roche). For crystal violet staining, cells were plated at 4 or 7.5 × 10^4^ in a 12-well plate, cultured for 7 to 14 days, and stained with crystal violet (Wako). For WST-1 assay, U2OS cells were plated at 5 × 10^3^ cells per well in a 96-well plate and cultured for 14 days, followed by the addition of WST-1 reagent to the medium and additional incubation for 20 min. The cleavage of the tetrazolium salt WST-1 by mitochondrial dehydrogenases was measured by absorbance at 450 nm.

### Statistical analysis

The two-tailed Student’s t-test was used to calculate *p*-values for all data sets.

## Data availability

All data are given in the main manuscript or supporting information.

## Conflict of interest

The authors declare that they have no conflicts of interest with the contents of this article.
