# Supplementary material for: LY6D-induced macropinocytosis as a survival mechanism of senescent cells
Source: J Biol Chem. 2020 Nov 24;296:100049. doi: 10.1074/jbc.RA120.013500 (PMC7948989; doi:10.1074/jbc.RA120.013500)
Supplement: Supplementary file 1 — Supplementary Figures [file mmc1.pdf]

LY6D-induced macropinocytosis as a survival mechanism of senescent cells

Authors: Taiki Nagano[1], Tetsushi Iwasaki[1,2,3], Kengo Onishi[2], Yuto Awai[3], Anju Terachi[2], Shione Kuwaba[3], Shota Asano[2], Ryoko Katasho[2], Kiyoko Nagai[1], Akio Nakashima[1,4], Ushio Kikkawa[1,4] and Shinji Kamada[1,2,3]

Affiliations: [1]Biosignal Research Center, Kobe University, 1-1 Rokkodai-cho, Nada-ku, Kobe 657-8501, Japan.
[2]Department of Biology, Graduate School of Science, Kobe University, 1-1 Rokkodai-cho, Nada-ku, Kobe 657-8501, Japan.
[3]Department of Biology, Faculty of Science, Kobe University, 1-1 Rokkodai-cho, Nada-ku, Kobe 657-8501, Japan.
[4]Department of Bioresource Science, Graduate School of Agricultural Science, Kobe University, 1-1 Rokkodai-cho, Nada-ku, Kobe 657-8501, Japan.

This PDF file includes Supporting Figures S1-S6.

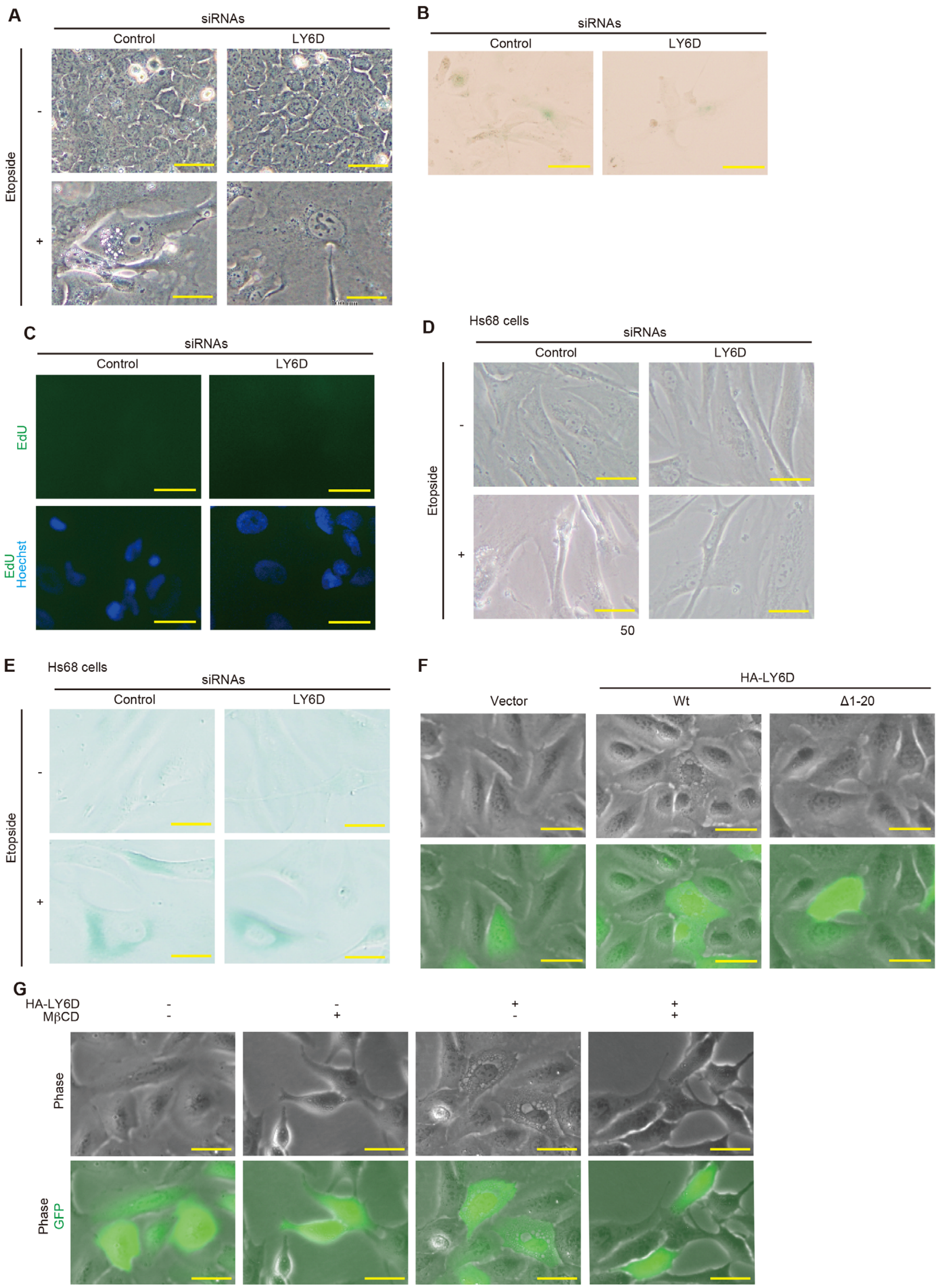

Figure S1. LY6D induces vacuole formation during senescence, which requires its localization to cell membrane rafts (representative microscopic images). *A-C*, U2OS cells transfected with siRNA for *LY6D* and treated with 2 μM etoposide for 7 days were subjected to

quantification of vacuole-forming cells (*A*), SA-β-gal staining (*B*), and EdU incorporation assay (*C*). *Bars*, 50 μm. *D* and *E*, Hs68 cells transfected with siRNA for *LY6D* and treated with 0.5 μM etoposide for 7 days were subjected to quantification of vacuole-forming cells (*D*) and SA-β-gal staining (*E*). *Bars*, 50 μm. *F*, U2OS cells transfected with pcDNA3-HA containing Wt and Δ1-20 *LY6D* were subjected to quantification of vacuole-forming cells. *Bars*, 50 μm. *G*, U2OS cells were overexpressed with HA-LY6D-Wt, treated with 2.5 mM MβCD for 17 h, and subjected to quantification of vacuole-forming cells. *Bars*, 50 μm.

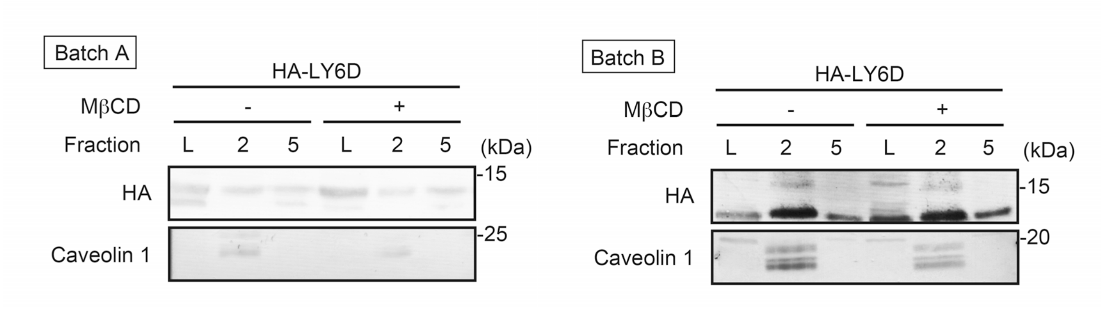

Figure S2. LY6D protein level in fraction 2 was reduced by the MβCD treatment. Lysates of LY6D-overexpressed U2OS cells treated with 2.5 mM MβCD for 17 h (two batches, Batch A and Batch B) were fractionated by sucrose density gradient centrifugation, and the raft-containing fraction (Fraction 2) and the non-raft fraction (Fraction 5) were subjected to immunoblot analysis. Caveolin 1 was used as a raft marker, indicating that Fraction 2 contained the raft fraction. Fraction L represents whole cell lysate.

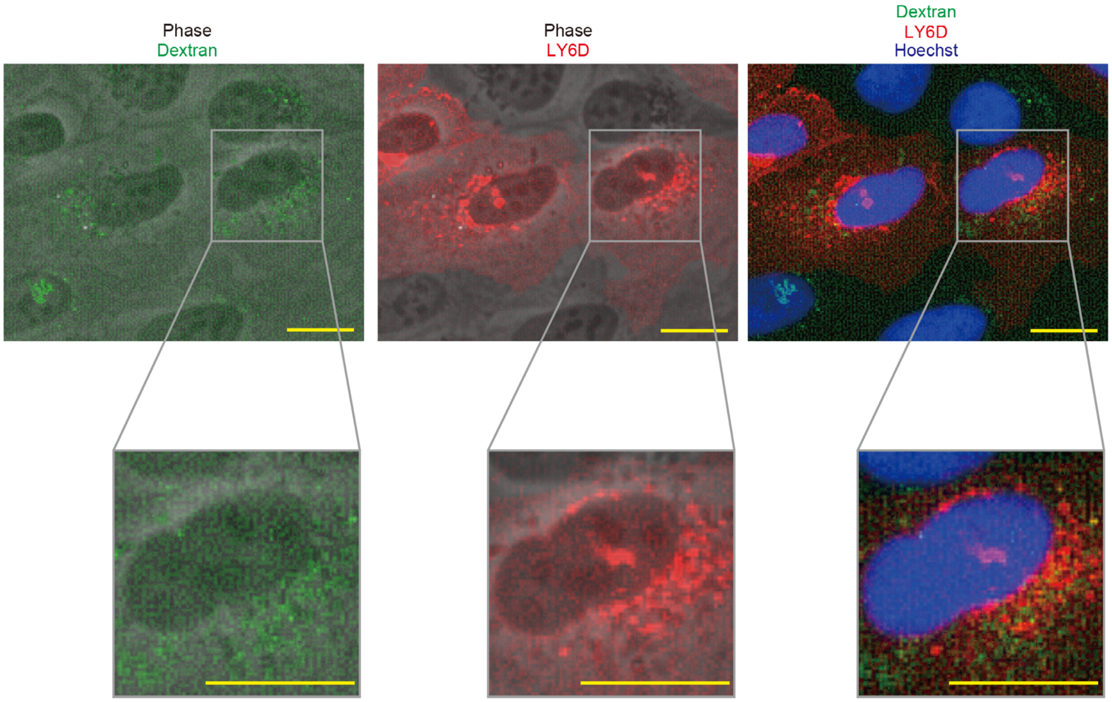

Figure S3. LY6D is not localized to the vacuoles. U2OS cells transfected with pcDNA3-HA containing *LY6D* and incubated with dextran-Alexa Fluor 488 (10,000 MW) for 16 h were subjected to immunostaining with the anti-HA antibody. *Bars*, 50 µm

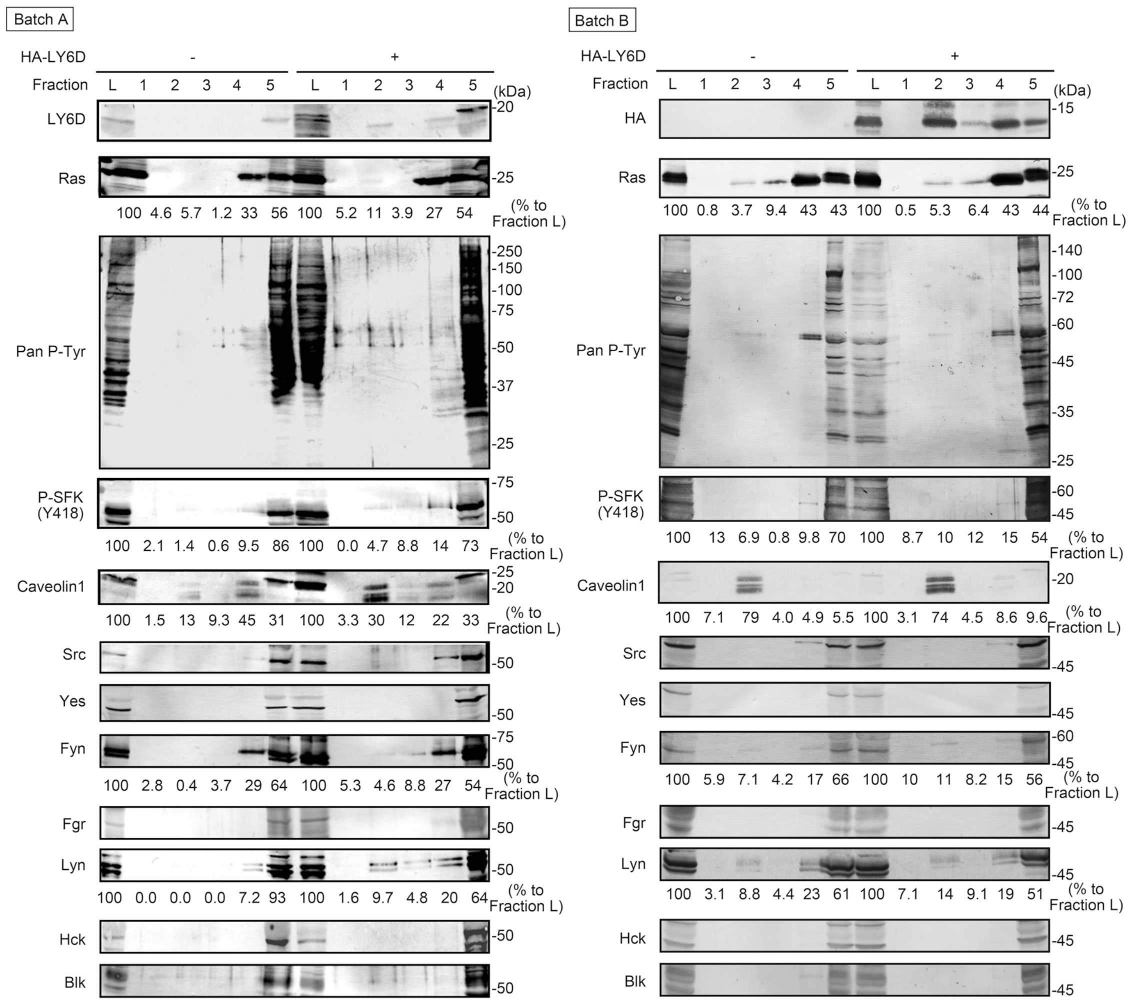

Figure S4. LY6D, Ras, and SFK accumulates in the raft fraction. Lysates of HA-LY6D-overexpressing U2OS cells (two batches, Batch A and Batch B) were fractionated by sucrose density gradient centrifugation. The obtained fractions 1 to 5 from the top (light fraction) to the bottom (heavy fraction) of the ultracentrifuge tube were subjected to immunoblot analysis. Caveolin 1 was used as a raft marker, indicating that Fraction 2 contained the raft fraction. Fraction L represents whole cell lysate. The protein levels of Ras, P-SFK, Caveolin 1, Fyn, and Lyn in each fraction were quantified by ImageJ software and shown as percentages to the fraction L.

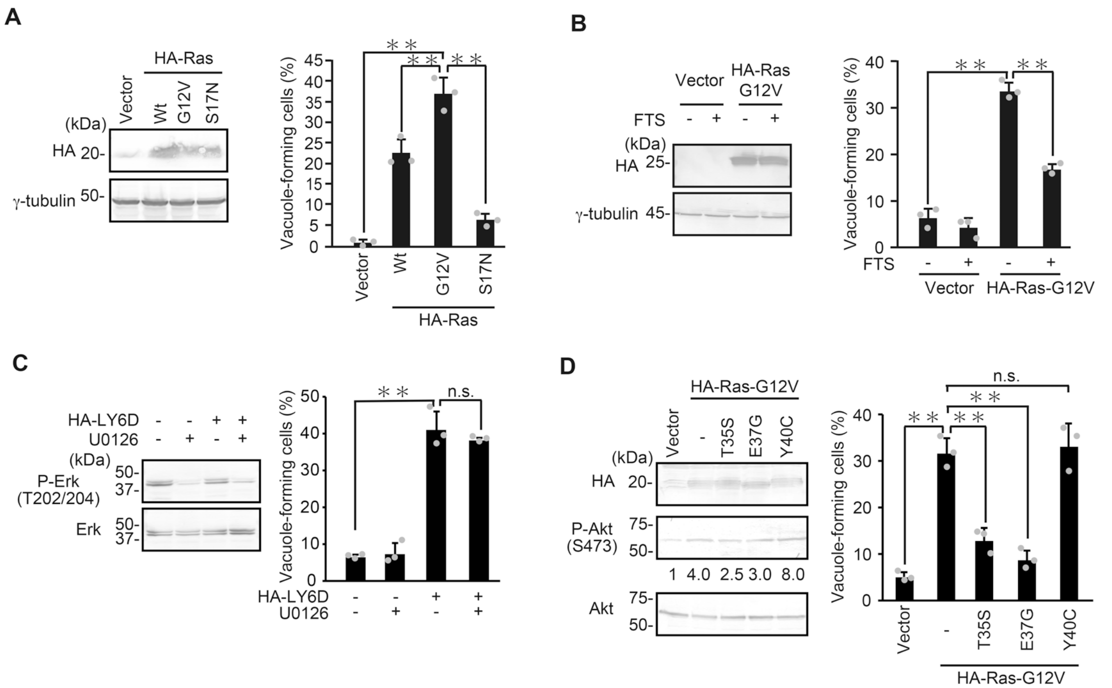

Figure S5. Ras induces vacuole formation through the PI3K pathway. *A*, U2OS cells transfected with pcDNA3-HA containing Wt-, G12V-, and S17N-Ras were subjected to immunoblot analysis (left panel) and quantification of vacuole-forming cells (right panel). *B*, U2OS cells pretreated with 100 μM FTS for 2 h were then transfected with pcDNA3-HA-Ras-G12V and subjected to immunoblot analysis (left panel) and quantification of vacuole-forming cells (right panel). *C*. HA-LY6D-overexpressing U2OS cells treated with 10 μM U0126 for 16 h were subjected to immunoblot analysis (left panel) and quantification of vacuole-forming cells (right panel). *D*, U2OS cells transfected with pcDNA3-HA containing *Ras* with the indicated point mutation (T35S, E37G, and Y40C, all in the G12V background) were subjected to immunoblot analysis (left panel) and quantification of vacuole-forming cells (right panel). The Akt phosphorylation levels relative to total Akt level were quantified using NIH ImageJ software and indicated at the bottom of each lane. Data are mean ± s.d. (n = 3 independent cultures). Statistical significance is shown using the Student's t-test analysis; $**P < 0.01$; n.s. = not significant ($P > 0.05$).

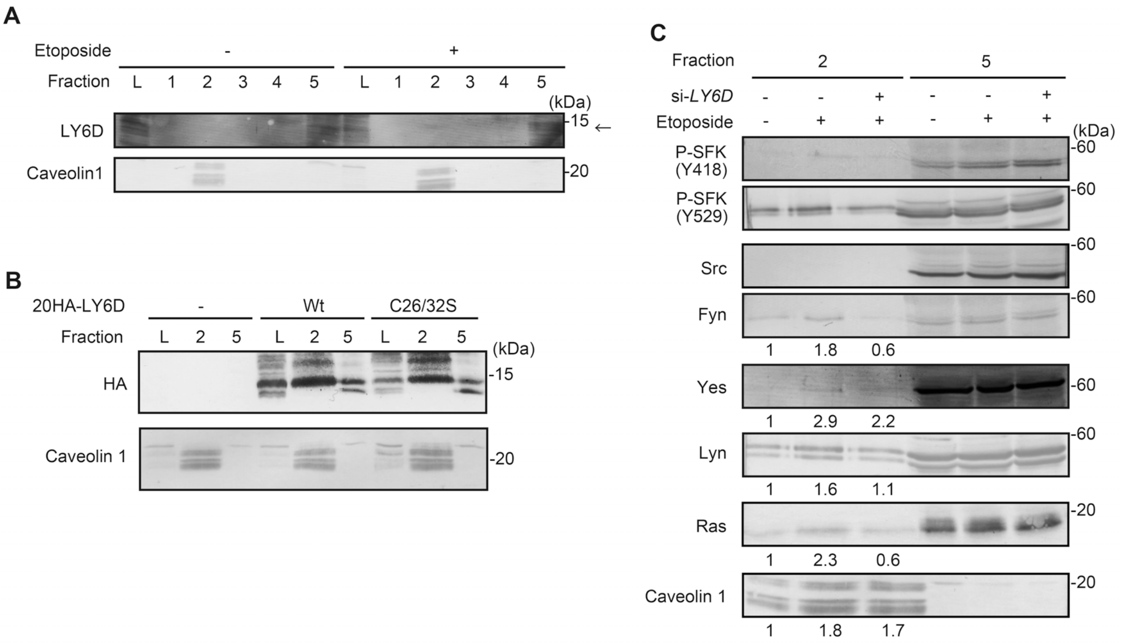

Figure S6. The results of different batch raft experiments. *A*, The results of different batch raft experiments related to Fig. 2*I*. Lysates of etoposide-treated U2OS cells were fractionated by sucrose density gradient centrifugation. The obtained fractions 1 to 5 from the top (light fraction) to the bottom (heavy fraction) of the ultracentrifuge tube were subjected to immunoblot analysis. Caveolin 1 was used as a raft marker, indicating that Fraction 2 contained the raft fraction. Fraction L represents whole cell lysate. *B*, The results of different batch raft experiments related to Fig. 5*D*. Lysates of U2OS cells overexpressed with 20HA-LY6D-Wt or C26/32S were fractionated by sucrose density gradient centrifugation, and the raft-containing fraction (Fraction 2) and the non-raft fraction (Fraction 5) were subjected to immunoblot analysis. *C*, The results of different batch raft experiments related to Fig. 5*E*. Lysates of LY6D-depleted U2OS cells treated with etoposide were fractionated by sucrose density gradient centrifugation, and the raft-containing fraction (Fraction 2) and the non-raft fraction (Fraction 5) were subjected to immunoblot analysis. The relative protein levels of Fyn, Yes, Lyn, and Ras in Fraction 2 normalized to the caveolin 1 level were determined using NIH ImageJ software and indicated at the bottom of each lane.
